# Bystander Intervention Efficacy to Reduce Teen Dating Violence Among High School Youth Who Did and Did Not Witness Parental Partner Violence: A Path Analysis of A Cluster RCT

**DOI:** 10.1007/s10896-021-00297-y

**Published:** 2021-06-29

**Authors:** Annelise Mennicke, Heather M. Bush, Candace J. Brancato, Ann L. Coker

**Affiliations:** 1 University of North Carolina at Charlotte, 9201 University City Blvd, Charlotte, NC 28223 USA; 2 University of Kentucky, Lexington, KY USA

**Keywords:** Bystander intervention, Parental IPV, High-risk youth, Dating violence victimization and perpetration

## Abstract

Youth who witness parental intimate partner violence (IPV) are at increased risk of teen dating violence (DV). This analysis of secondary data investigated whether a bystander intervention program, *Green Dot,* was effective at reducing physical and psychological DV victimization and perpetration among youth who had and had not previously witnessed parental IPV. The parent RCT assigned 13 schools to control and 13 schools to the *Green Dot* intervention. Responses from 71,797 individual surveys that were completed by high school students were analyzed across three phases of a 5-year cluster randomized control trial. Multigroup path analyses revealed that students in intervention schools who witnessed parental IPV had a reduction in psychological (*p* < .001) and physical DV (*p* < .01) perpetration and psychological DV victimization (*p* < .01) in Phase 2 of the intervention, while those who did not witness parental IPV had a significant reduction in psychological DV victimization (*p* < .01). Individuals in the intervention received more training (*p* < .001), which was associated with lower levels of violence acceptance (*p* < .001). Violence acceptance was positively associated with DV victimization and perpetration (*p* < .001), especially for individuals who previously witnessed parental IPV. *Green Dot* is an effective program at reducing DV victimization and perpetration among the high-risk group of youth who previously witnessed parental IPV, largely operating through violence acceptance norms. This underscores the bystander intervention approach as both a targeted and universal prevention program.

## Introduction

An alarming number of both female and male youth experience teen dating violence (DV) (Basile et al., 2020; Hamby & Turner, 2013; Kann et al., 2014; Taylor & Mumford, 2016; Vagi et al., 2015). Teen DV is defined as physical violence, sexual violence, psychological aggression, and stalking between two teenagers in a close relationship (Breiding et al., 2015). Experiencing teen DV is associated with a host of negative consequences, ranging from trauma symptoms, coping behaviors such as substance use, and negative academic impacts (Ackard et al., 2007; Banyard & Cross, 2008; Wolfe, et al., 2003a, b). Witnessing family or parental intimate partner violence (IPV) has been identified as the strongest risk factor for adult IPV victimization (Park & Kim, 2018). Violence acceptance, which herein is defined as acceptance of dating violence and endorsement of rape myths, is higher among individuals who have been exposed to family violence (Karlsson et al., 2019; Kinsfogel & Grych, 2004), and has been found to mediate the relationship between violence exposure and violence victimization/perpetration (Lee et al., 2016; Lichter & McCloskey, 2004; Marciniak, 1998; Maxwell et al., 2003). Thus, with one in four youth having ever witnessed parental IPV (U.S. Department of Health and Human Services, 2019), finding effective interventions for these higher risk youth is strategically important for reducing teen DV and IPV.

Bystander intervention programs are one strategy that has been identified as a promising approach to prevent sexual violence (Degue et al., 2014). The focus of bystander intervention programs is to train bystanders to take action to prevent potential harm from occurring, typically teaching skills along the five steps of bystander intervention (Latané & Darley, 1968). A bystander must be taught to see a situation, recognize it as problematic, feel responsible to do something about it, know what to do to disrupt it, then take action to try to prevent/stop it. While largely developed and tested to prevent sexual violence (Kettrey & Marx, 2019; Mujal et al., 2019), some bystander intervention programs have been demonstrated to reduce teen DV and also reduce harmful attitudes associated with teen DV (Coker et al., 2017; Edwards et al., 2019). This approach is in contrast to other universally-implemented prevention techniques which focus exclusively on teen DV by working to develop healthy relationship skills (e.g., Fourth-R and Safe Dates).

Because of emerging evidence that bystander programs are also effective at reducing teen DV in addition to sexual violence (Coker et al., 2017; Edwards et al., 2019), it is likely that these bystander intervention programs, which are often universally targeted to general populations, work well among the high risk group of youth who were previously exposed to parental IPV. The purpose of this investigation is to assess whether one such universally-implemented bystander intervention program, *Green Dot*, reduces teen DV among youth exposed to parental IPV. In addition, we sought to examine how *Green Dot* works by assessing the mediating roles of amount of training received and violence acceptance. This is a secondary analysis of data which has previously examined the effectiveness of *Green Dot* using general high school samples. This investigation makes a unique contribution by stratifying the sample into two groups (those who witnessed parental IPV and those who did not witness parental IPV) to examine whether and how *Green Dot* works to reduce teen DV. Additionally, this investigation focuses on the individual-level data, whereas previous investigations using these data have assessed the effectiveness of *Green Dot* to prevent violence at the school level. Because the intevention is implemented at the school-level, prior analyses have created school-level scores by averaging responses from individuals. Because our interest is on the influence of the individual-level variable of witnessing parental IPV, we use individual-level variables throughout.

### Literature Review

There have been many efforts and some successes in reducing teen DV, with some focus on the high risk group of youth who were previously exposed to violence. Three main approaches will be reviewed here: 1) programs which aim to reduce teen DV specifically among the high risk group of youth who have been exposed to violence; 2) universally implemented traditional teen DV prevention/intervention programs; and 3) universally implemented bystander intervention programs.

#### Reducing Teen DV Among Youth with Violence Exposure

No known program specifically aims to reduce teen DV among youth who previously witnessed parental IPV. Several programs exist and have been evaluated to reduce violence among youth who have been exposed to other forms of violence. Researchers found that Expect Respect Support Groups were associated with a reduction of multiple types of teen DV, including psychological DV victimization and perpetration, sexual DV victimization and perpetration, and physical DV victimization among youth who had been exposed to violence in their home, school, or community (Reidy et al., 2017). A core component of Expect Respect Support Groups is to modify maladaptive norms about dating behavior. Another study found that a program called Date SMART was effective at reducing sexual DV involvement among girls who had previously been exposed to physical DV themselves (Rizzo et al., 2018). This intervention uses a cognitive-behavioral framework to disrupt maladaptive congitions and behaviors associated with DV risk. A community-implemented program called Youth Relationship Project, which targets youth who had previous exposure to child maltreatment, found significant reductions in teen DV perpetration (Wolfe, et al., 2003a, b). This program provides alternatives to aggresion-based problem solving and challenges gender-based role expectations. From this, we can see that there are reductions in teen DV outcomes associated with interventions that target youth who have been exposed to violence, and a commonality among these programs is challenging problematic social norms.

#### Universal Interventions to Reduce Teen DV

A number of intervention/prevention programs have been evaluated to reduce teen DV among more universal populations. Among high school students, support exists for the effectiveness of Fourth-R to reduce physical DV perpetration (Wolfe et al., 2009) and Safe Dates to reduce psychological and physical DV perpetration and physical DV victimization (Foshee et al., 2005). Among middle school students, Dating Matters has been shown to to reduce teen DV victimization and perpetration (Niolon et al., 2019), and Shifting Boundaries was associated with a reduction in sexual DV victimization (Taylor et al., 2013). Coaching Boys Into Men was associated with a reduction in teen DV perpetration (Miller et al., 2013). However, a meta-analysis summarizing the effectiveness of this primary teen DV prevention approach found null results on teen DV perpetration across three studies at post-test and follow-up, and small but significant reductions in teen DV victimization at post-test that became null at follow-up (De La Rue et al., 2014). These teen DV prevention approaches tend to use a mix of intervention strategies, including healthy relationship skills training and socio-emotional approaches, with elements of bystander intervention incorporated within them (Niolon et al., 2017). Notably, many programs demonstrate success in improving knowledge and reducing violence acceptance (Antle et al., 2011; Avery-Leaf et al., 1997; De La Rue et al., 2014; Jaffe et al., 1992; Jaycox et al., 2006; Jones, 1998; Lavoie et al., 1995; Ting, 2009), which may be an important mediator to influencing violence outcomes.

#### Universally Targeted Bystander Intervention Programs

Even more broad is the category of universally-targeted bystander intervention programs, which often focus on reducing sexual violence (defined as sexual activity when consent is not obtained or not freely given, and refers to acts that are perpetrated by anyone, including but not limited to a friend, current or former intimate partner, coworker, neighbor, or family member [Basile et al., 2014]), often among college students (Bell et al., 2019; Kettrey & Marx, 2019, 2020; Sargent et al., 2017). Guided by the situational model of bystander intervention (Burn, 2009; Latané & Darley, 1968), bystander programs attempt to train students to recognize potentially problematic situations and acquire the skills needed to helpfully intervene to prevent violence from occurring. Therefore, individuals who are trained in bystander intervention should demonstrate an increase in the number of bystander behaviors they use, which in turn should lead to a reduction in the number of violent incidents at the community/school-level (Burn, 2009). However, syntheses of evaluations of bystander intervention programs indicate null or mixed effects on sexual violence outcomes (Kettrey & Marx, 2019; Mujal et al., 2019). However, evaluations of two programs indicate that the bystander approach may be useful at reducing DV. For example, an RCT evaluation of *Green Dot* in high schools found a significant reduction in perpetration and victimization of physical and psychological DV at the school-level (Coker et al., 2017). Bringing in the Bystander has also demonstrated an ability to reduce DV victimization and perpetration rates in the past two months among high school youth (Edwards et al., 2019).

#### Mediators of Bystander Intervention Effectiveness

Several factors appear to mediate the relationship between bystander intervention programs and violence outcomes (generally sexual violence). Bystander programs have been found to reduce violence acceptance (Kettrey & Marx, 2019; Mujal et al., 2019), which is important because violence attitudes are associated with teen DV victimization and perpetration (Marciniak, 1998; Maxwell et al., 2003), and may be especially important among youth who have previously been exposed to parental IPV because they have higher rates of violence acceptance (Karlsson et al., 2019; Kinsfogel & Grych, 2004). Additionally, using school-level data from the same project as the current investigation, researchers found that violence acceptance and bystander behaviors mediate the relationship between intervention received at the school and sexual violence perpetration rates at the school-level (Bush et al., 2019). Thus, it is likely that violence acceptance acts as a mediator between receipt of bystander intervention training and individual-level teen DV victimization and perpetration, especially among youth who previously witnessed parental IPV.

As noted throughout, previous investigations using the parent data have focused on school-level analyses, as *Green Dot* is conceptualized as a community-level intervention designed to increase bystander behaviors among those trained, which will then be utilized among their peers to reduce interpersonal violence (Cook-Craig et al., 2014). However, as previously demonstrated, *Green Dot* also works to reduce violence acceptance (Coker et al., 2018, 2020), which mediates the relationship between the intervention and violence outcomes (Bush et al., 2019). Because previous investigations using *Green Dot* data within high schools have focused on the school-level data, there has not been an investigation into amount of training received by individuals, and how this may influence violence acceptance and violence outcomes among individuals. Investigations into amount of *Green Dot* training received among college students indicates that individuals who receive more training had lower rape myths (Coker et al., 2011). Therefore, it is likely that high school students who receive more training have lower levels of violence acceptance. Furthermore, because youth who have been exposed to parental IPV have higher levels of violence acceptance (Karlsson et al., 2019; Kinsfogel & Grych, 2004), we hypothesize that there will be greater reductions in violence acceptance among this high-risk group.

#### Conceptual Model

**Fig. 1:**
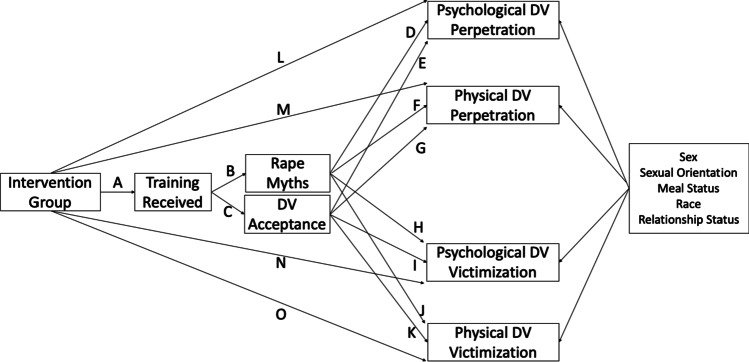
Conceptual model for Phase 1 and Phase 2

We used the following conceptual model to guide our analyses (Fig. 1). In this model, the intervention is theorized to be associated with amount of training received (path A). Amount of training is hypothesized to be related to endorsement of rape myths and DV acceptance (paths B and C). Rape myths and DV acceptance are expected to be associated with violence outcomes (Paths D-K). Finally, school-level intervention status is expected to be associated with individual-level violence outcomes (Paths L-O). We did not estimate a direct path between intervention and rape myths or DV acceptance, as we would not expect that being in an intervention school but not receiving the training individually would lead to changes in violence norms within a given year/phase. Furthermore, we did not estimate a direct path from training received to violence outcomes because the training primarily focuses on increasing bystander behaviors at the school-level to reduce violence and does not directly address individual-level risk reduction or perpetration reduction techniques.

## Research Questions

Prior investigations have not examined the efficacy of universal bystander approaches for the high-risk group of youth who have previously witnessed parental IPV, nor have they examined the potential mediating role of training received and violence acceptance among this group. This investigation aims to add knowledge to these gaps by answering two research questions.

- Among students who witnessed parental IPV, was receipt of *Green Dot* bystander training associated with lower frequencies of physical and psychological teen DV victimization or perpetration compared to students who did not receive training?
- Among students who did not witness parental IPV, was receipt of *Green Dot* bystander training associated with lower frequencies of physical and psychological teen DV victimization or perpetration compared to students who did not receive training?
- Among students who witnessed parental IPV, does training received, endorsement of rape myths and DV acceptance mediate the relationship between intervention status and DV perpetration and victimization?
- Among student who did not witness parental IPV, does amount of training received, endorsement of rape myths, and DV acceptance mediate the relationship between intervention status and DV perpetration and victimization?

## Methods

### Design

This study is a secondary analysis of data from a five-year cluster randomized control trial evaluating the effectiveness of *Green Dot* (Clear et al., 2014; Coker et al., 2017). Prior evaluations using these data have found that *Green Dot*, at the school-level, significantly reduces perpetration and victimization of physical and psychological teen DV (Coker et al., 2017), and that effectiveness of the intervention to reduce sexual violence perpetration is mediated by violence acceptance and bystander behaviors (Bush et al., 2019). However, these examinations have not yet been examined among the high-risk group of youth who previously witnessed parental IPV. Bystander behaviors were not included in these analyses because we would not expect that there would be an association between bystander behaviors and violence outcomes at the individual-level.

The parent study selected 26 high schools in Kentucky to participate in a randomized intervention trial to evaluate the effectiveness of a bystander intervention program, *Green Dot*, over 5-years (Coker et al., 2017). Randomization occurred at the school-level, with 13 schools randomized to the treatment condition and 13 schools randomized to the control condition.

### Procedure

Data were collected by inviting all students to complete an anonymous in-person survey from 2010 through 2014. Researchers coordinated with schools to identify two days between February and April each year to come to the school and collect data. Parents could have their children opt out of the survey. Assent was gathered from the student before passing out the survey. The survey was a 99-item paper and pencil questionnaire administered by research staff which took 20–45 min to complete.

### Intervention

The study period was divided into three distinct phases defined by the degree of intervention implementation in schools randomized to receive *Green Dot*. Phase 0 represents Year 1 (baseline) of the study, where no intervention was implemented in either condition. In Phase 1 (years 2 and 3) of intervention implementation, more than 50% of students in the intervention schools received 50-min introductory persuasive speeches (also called overview speeches) delivered by educators from local rape crisis centers. Details about the components of the intervention can be found in Coker et al. (2011). Briefly, the overview speech introduces students to the concept of helpful bystander behaviors, increases knowledge and challenges myths related to interpersonal violence, and creates a shared sense of community that everyone has a role to play in stopping violence. These overview speeches were provided annually to students in the intervention schools and students could participate in multiple overview speeches throughout the years. In Phase 2 (years 4 and 5) of the intervention, key student influencers (10–15% of the student body) participated in a five-hour intensive skills-based training. This training focused on raising awareness of red flags for violence and breaking down barriers to inaction by focusing on the “three D’s” of bystander intervention: delegate, distract, or directly intervene. Key students were identified by staff at the intervention high schools. While phases are defined by study years, schools randomized to the control condition continued programming as usual, with research staff ensuring that no bystander intervention program was implemented during this time (Coker et al., 2017).

### Participants

**Fig. 2:**
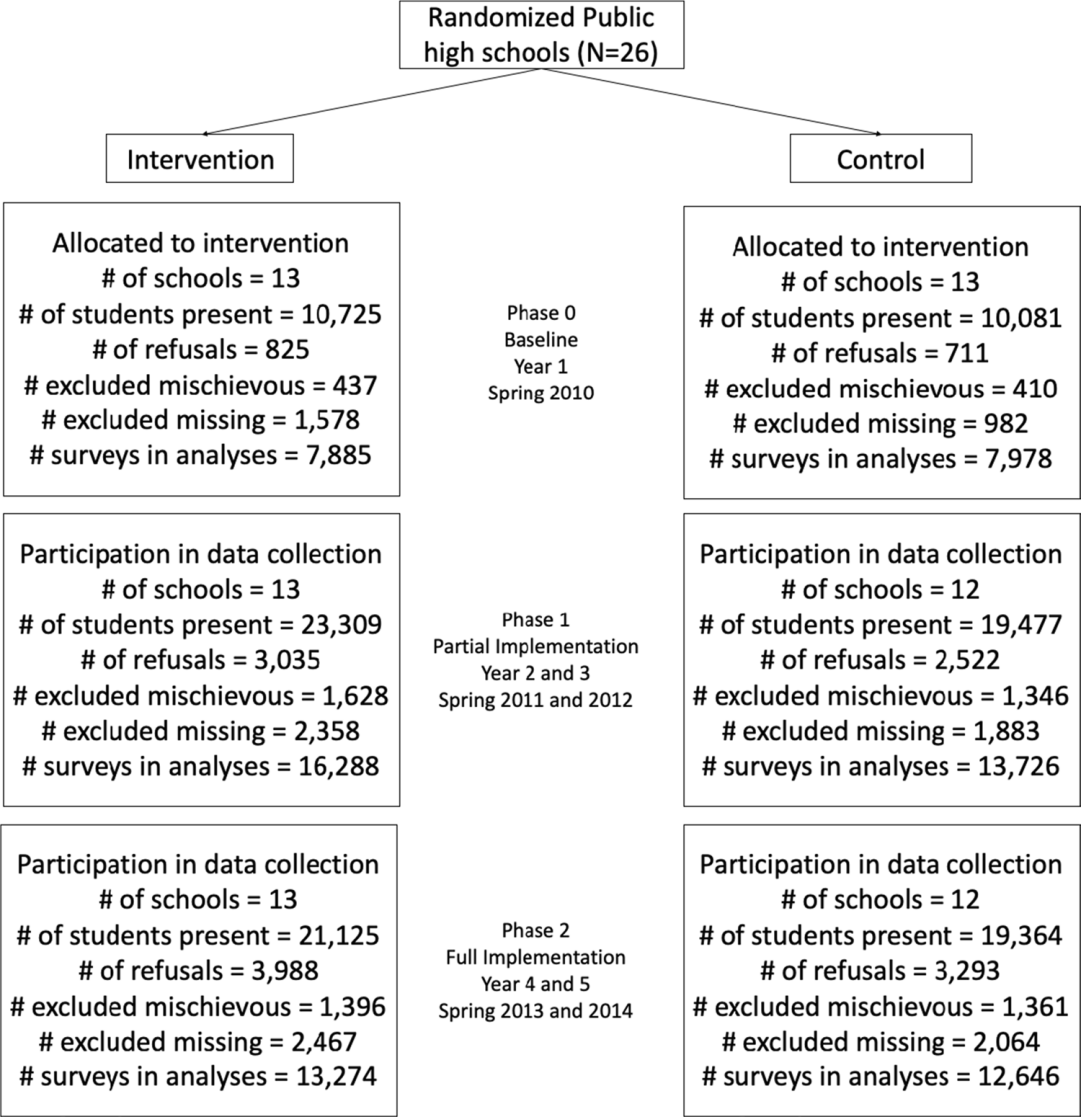
CONSORT diagram

Participants in the parent study were high school youth between the ages of 14–18 who completed annual surveys. A total of 89,707 surveys were returned from students across the study period, but because the surveys were anonymous and the intervention was implemented at the school-level, individual students are not linked across years. From the parent study, data were limited to individuals who were not missing information on all demographic variables nor violence or intervention training items. Additionally, surveys were excluded if they contained mischievous responses (see Coker et al., 2017 for additional details). This resulted in a data set containing 74,878 surveys. For the current analysis, additional surveys were excluded if there was data missing on any of the variables of interest (n = 3,094, 4.1%). An analysis comparing survey completers to non-completers was conducted and found that a greater proportion of survey non-completers were in higher grades, received a free or reduced-priced meal, experienced family abuse, and were male, non-White, and not exclusively heterosexual compared to survey completers (*p* < 0.001). However, multiple imputation was not utilized to replace missing variables due to the dubious accuracy of predicting scores on behavioral outcomes like violence victimization and perpetration and the small amount of missingness. The analytic sample for this analysis contained n = 71,797 surveys (see Fig. 2 for a CONSORT diagram).

The sample for the current study consisted of 57% of individuals who identified as female, 30% were in the 9^th^ grade, 83% were White, 45% received a free or reduced price lunch, 86% indicated their sexual orientation as exclusively heterosexual, 21% witnessed parental IPV, and 78% indicated they had been in a relationship in the past 12 months (see Table 1 for details). Just over half (52%) of the sample came from intervention schools, and the modal group from the sample responded to surveys during Phase 1 of the intervention (41.8%).

**Table 1:** Sample characteristics by intervention phase for all students and between student who did and did not witness parental IPV

	All Phases N = 71,797			Phase 0 n = 15,863			Phase 1 n = 30,014			Phase 2 n = 25,920		
	All Surveys	Did Not Witness	Did Witness Parental IPV	All Students	Did Not Witness Parental IPV	Did Witness Parental IPV	All Students	Did Not Witness Parental IPV	Did Witness Parental IPV	All Students	Did Not Witness Parental IPV	Did Witness Parental IPV
Variable	%	%	%	%	%	%	%	%	%	%	%	%
Gender					**			**			**	
Female	56.5	54.8	63.1	54.6	52.5	60.9	56.3	54.5	63.0	58.1	56.5	65.1
Male	43.5	45.2	36.9	45.4	47.5	39.1	43.7	45.5	37.0	41.9	43.5	34.9
Grade								**			**	
9^th^	29.7	29.2	31.5	30.3	30.2	30.7	28.9	28.3	31.1	30.2	29.6	32.8
10^th^	27.6	27.5	27.8	27.7	27.6	27.9	27.2	27.2	17.2	28.0	27.8	28.5
11^th^	24.5	24.8	23.7	25.5	25.5	25.8	24.6	24.9	23.6	23.8	24.2	22.1
12^th^	18.0	18.4	16.5	16.3	16.6	15.4	19.0	19.4	17.6	17.9	18.2	16.1
Non-White race	17.1	15.7	22.4	16.3	14.9**	20.5	17.8	16.3**	23.4	16.8	15.4**	22.8
Free or reduced-price meal	44.8	40.7	60.7	42.8	37.2**	59.7	43.3	39.1**	59.4	52.2	44.4**	63.1
Not exclusively heterosexual	14.0	11.9	22.2	13.3	11.0**	20.0	13.6	11.4**	21.9	15.0	12.9**	24.3
Witnessed parental IPV	20.8			24.9			20.7			18.5		
Relationship in past 12 months	77.7	75.8	84.9	80.7	78.8**	86.3	78.9	77.1**	85.7	74.5	72.6**	82.6
Intervention Group	52.2	52.4	51.1	49.7	49.9	49.2	54.3	54.3	54.2	51.2	51.8**	48.8
Amount of Training					*			**			**	
Control School	47.8	47.6	48.9	50.3	50.1	50.8	45.7	45.7	45.8	48.8	48.2	51.2
Intervention—no training	29.8	29.4	31.2	49.1	49.4	48.2	23.1	22.7	24.9	25.7	25.8	25.4
Intervention – Overview speech	18.9	19.4	17.1	0.5	0.4	0.8	26.8	27.2	25.4	21.0	21.3	19.7
Intervention –2 + hours of training	3.5	3.6	2.8	0.1	0.0	0.2	4.3	4.4	3.9	4.5	4.7	3.7

The All Phases column represents all survey responses across study years. As surveys were anonymously collected, surveys may repeat across phases. Summaries are descriptive; statistical tests were only performed within phases where number of surveys equated to number of individuals. “All Students” refers to the number of student surveys collected within each phase

^*^ = significantly different from the did witness parental IPV group at *p* < .01

^**^ = significantly different from the did witness parental IPV group at *p* < .001

### Materials

Detailed information about the materials used, including psychometric information, can be found in Cook-Craig et al. (2014). All measures demonstrated acceptable reliability statistics, with alpha α > 0.70.

### Grouping Variables

#### Intervention Group

Students were marked as attending a school randomized to the intervention or control condition.

#### Witnessed Parental IPV

One question ascertained whether the student had witnessed parental IPV. It read, “In your family how often did you see or hear one of your parents or guardians being hit, slapped, punched, shoved, kicked, or otherwise physically hurt by their spouse or partner?” Response options included: never, 1 time, 2–5 times, 6–10 times, or more than 10 times. This variable was dichotomized to indicate those who have never witnessed parental IPV (i.e., response option never) and those who had witnessed parental IPV (i.e., 1 time or more response options) to allow for stratification.

### Violence Outcome Variables

#### Psychological DV

Three questions were used to assess for the amount of psychological DV experienced in a relationship (for each domain of victimization and perpetration). These items were revised from the National Intimate Partner and Sexual Violence Survey (Black et al., 2011). These questions included whether the student was a victim of or perpetrated the following behaviors: 1) Tried to control you by always checking up on you, telling you who your friends could be, or telling you what you could do and when; 2) Damaged something that was important to you on purpose; or 3) Threatened to hit, slap, or physically hurt you. Response options included: 0 times; 1–2 times; 3–5 times; 6–9 times; 10 or more times; yes this happened before but not in the past 12 months; or not in a dating or a romantic relationship in the past 12 months. Due to a data collection error one year in which the full response options were not provided, responses were recoded into the following categories: 0 times (including 0 times, those who indicated it had not happened in the past 12 months, and those who were not in a relationship); 1 time, 3 times, or 6 or more times, resulting in a potential range of 0–18.

#### Physical DV

One question was used to determine the frequency of physical DV victimization or perpetration. These items were revised from the National Intimate Partner and Sexual Violence Survey (Black et al., 2011). The questions asked individuals to indicate the number of times they or their partner “hit, slapped, or physically hurt you on purpose”. Response options included: 0 times; 1–2 times; 3–5 times; 6–9 times; 10 or more times; yes this happened before but not in the past 12 months; or not in a dating or a romantic relationships in the past 12 months. Due to a data collection error one year, responses were recoded into the following categories: 0 times (including 0 times, those who indicated it had not happened in the past 12 months, and those who were not in a relationship); 1 time, 3 times, or 6 or more times to make variables consistent across years, resulting in a potential range of 0–18.

### Mediating variables

#### Rape Myths

Seven questions taken from a modified version of the Illinois Rape Myth Acceptance Scale (Payne et al., 1999) were used to assess the student’s endorsement of rape myths. Response options ranged from 0 = strongly disagree to 3 = strongly agree. Based on a confirmatory factor analysis of this scale in this sample, one item was deleted from this scale:

“Girls lead a guy on and then they claim sexual assault,” resulting in a 6-item scale with acceptable fit statistics (RMSEA = 0.04, SRMR = 0.02, CFI = 0.98). Scores on this summed measure had a potential range of 0–18, with higher scores indicating greater endorsement of rape myths.

#### DV Acceptance

Five items taken from the Acceptance of General Dating Violence subscale of the Acceptance of Couple Violence scale (Foshee et al., 1998) were used to assess the student’s acceptance of DV. Response options included 0 = strongly disagree to 3 = strongly agree. A sum of these items was tabulated, yielding a potential range of 0–15, with higher scores indicating greater acceptance of DV.

#### Training Received

Individuals were asked to indicate how much *Green Dot* training they received. Individuals from the control school were coded as 0. Individuals from intervention schools who indicated they did not receive any training were coded as 1. Individuals from the intervention school who indicated that they received only a *Green Dot* overview speech were coded as 2. Individuals from the intervention school who heard a *Green Dot* overview speech within the last year and who received 2 + hours of training were coded as 3.

### Demographic Control Variables

#### Sex

Students were asked to report whether their sex was female or male.

#### Sexual Orientation

Students were asked to report their sexual attraction. Responses included “only attracted to females,” “mostly attracted to females,” “equally attracted to females and males,” “mostly attracted to males,” “only attracted to males,” and “not sure.” Students were categorized as indicating heterosexual (including only attracted to females for the male respondents and only attracted to males for the female respondents) or not exclusively heterosexual (including all the responses not captured in the heterosexual group).

#### Meal Status

Students were asked to indicate whether they received a free or reduced meal at school (yes or no), which is a proxy for income.

#### Race

Students were asked to indicate their racial identification. Categories included White, American Indian or Alaska native, Asian, Black or African American, or Hispanic or Latino/Latina. These were dichotomized into Non-Hispanic White and non-White.

#### Relationship Status

Individuals were asked to indicate if they had been in a dating relationship in the past 12 months. Responses were dichotomized to indicate 0 = they were not in a relationship during the past 12 months or 1 = they were in a relationship in the past 12 months.

#### Analytic Approach

Study characteristics and variables of interest (e.g., violence acceptance and violence outcomes) were summarized overall (all surveys combined across all years) and within intervention phase. Within each intervention phase, students reporting witnessing parental IPV were compared to students who did not witness parental IPV using chi-square tests of independence and t-tests.

To estimate pre-intervention/training relationships, the path analyses were constructed for Phase 0; analyses conducted for Phases 1 and 2 examined these relationships and the association between training received on violence acceptance, and violence acceptance on violence outcomes. We utilized a path analysis approach but did not use multilevel modeling to account for school-level clustering because of the extremely low intraclass correlation values observed (ICC ≤ 0.006), indicating that there was not a clustering effect on violence outcomes that needed to be accounted for. Frequencies of victimization and perpetration were adjusted for by including paths between the following demographic controls and violence outcomes: sex, sexual orientation, meal status, race, and relationship status, based on prior research finding associations between victimization/perpetration and these demographic characteristics (Clear et al., 2014). To test the conceptual model and variable relationships, multigroup path analyses (estimating model fit and paths for two groups at once) were used to examine how the intervention operates at the individual-level between those who witnessed parental IPV and those who did not witness parental IPV. Because individuals are not linked over time, the path analyses were conducted separately for each phase. Path models for all students within a phase (model fit statistics and paths estimated for the sample as one group) were constructed first, and the multigroup model was retained if the chi-square difference test indicated it was a better fit to the data (*p* < 0.01). Models were considered to be a good fit to the data if RMSEA and SRMR were equal to or less than 0.1 and if CFI was equal to or greater than 0.90 (Kline, 2010). Typical chi-square tests (*p* > 0.05) were not used to assess overall model fit because these tests are sensitive to sample size and the sample size for these analyses were quite large. However, the chi-square difference test is a reliable indicator when comparing between models, as both models are similarly affected by the large sample size.

Path analysis results are presented in a table with unstandardized regression coefficients, corresponding *p*-values, and 99% confidence intervals (CI) of the unstandardized beta. Results for the path analysis at Phase 2 are additionally presented graphically. A two-sided significance level of 0.01 was used for all statistical tests because of the size of the sample. Data management, and descriptive/bivariate analyses were completed using SPSS version 26 and path analyses were completed using Amos version 26.

## Results

### Descriptive and Bivariate Statistics

Table 1 contains the descriptive statistics for the full sample as well as the subsamples within each phase of the intervention. Demographic characteristics of the subsamples within each phase were similar to the full sample, described above. Chi-square tests and Fishers Exact Tests revealed significant differences on all demographic variables within each phase. In general, a higher proportion of students who witnessed parental IPV reported being female, in 9^th^ grade, non-White, not exclusively heterosexual, in a relationship in the past 12 moths, and receiving a free or reduced-price meal compared to students who did not witness parental IPV.

Table 2 contains the descriptive statistics for the outcome variables of interest for the full sample as well as subsamples within each phase of the intervention. Psychological DV victimization was the most frequently reported violence outcome whereas physical DV perpetration was the least frequently reported violence outcome. Across phases, individuals who witnessed parental IPV had higher levels of rape myths, were more accepting of DV, and had higher frequencies of all forms of DV victimization and perpetration as indicated by significant t-tests.

**Table 2:** Variables of interest by intervention phase for all students and between student who did and did not witness parental IPV

	All Phases N = 71,797			Phase 0 n = 15,863			Phase 1 n = 30,014			Phase 2 n = 25,920		
Variable	All Surveys M (SD)	Did Not Witness Parental IPV M (SD)	Did Witness Parental IPV M (SD)	All Students M (SD)	Did Not Witness Parental IPV M (SD)	Did Witness Parental IPV Parental IPV M (SD)	All Students M (SD)	Did Not Witness Parental IPV M (SD)	Did Not Witness Parental IPV M (SD)	All Students M (SD)	Did Witness Parental IPV M (SD)	Did Witness Parental IPV M (SD)
Rape Myths	4.31 (3.02)	4.15 (2.85)	4.94 (3.53)	4.71 (2.90)	4.62** (2.81)	4.97 (3.17)	4.35 (3.05)	4.18** (2.87)	4.98 (3.59)	4.02 (3.02)	3.83** (2.81)	4.84 (3.72)
DV Acceptance	2.73 (2.79)	2.53 (2.60)	3.48 (2.33)	3.15 (2.74)	3.00** (2.62)	3.60 (3.03)	2.68 (2.83)	2.47** (2.63)	3.45 (3.39)	2.52 (2.75)	2.31** (2.51)	3.42 (3.48)
Psychological DV perpetration	0.57 (2.03)	0.37 (1.45)	1.30 (3.33)	0.58 (1.90)	0.41** (1.44)	1.08 (2.83)	0.60 (2.12)	0.40** (1.54)	1.38 (3.44)	0.52 (1.99)	0.32** (1.34)	1.37 (3.56)
Physical DV perpetration	0.16 (0.78)	0.09 (0.57)	0.40 (1.27)	0.16 (0.77)	0.11** (0.60)	0.34 (1.13)	0.17 (0.81)	0.10** (0.59)	0.44 (1.32)	0.14 (0.74)	0.08** (0.52)	0.41 (1.31)
Psychological DV victimization	1.37 (3.05)	1.02 (2.49)	2.70 (4.35)	1.47 (3.06)	1.12** (2.56)	2.54 (4.06)	1.42 (3.12)	1.05** (2.54)	2.82 (4.44)	1.25 (2.97)	0.93** (2.39)	2.68 (4.47)
Physical DV victimization	0.25 (1.01)	0.16 (0.80)	0.60 (1.54)	0.27 (1.03)	0.19** (0.85)	0.51 (1.41)	0.27 (1.05)	0.17** (0.82)	0.64 (1.58)	0.23 (0.96)	0.14** (0.73)	0.61 (1.58)

The All Phases column represents all survey responses across study years. As surveys were anonymously collected, surveys may repeat across phases. Summaries are descriptive; statistical tests were only performed within phases where number of surveys equated to number of individuals. “All Students” refers to the number of student surveys collected within each phase

^*^ = significantly different from the did witness parental IPV group at *p* < .01

^**^ = significantly different from the did witness parental IPV group at *p* < .001

### Path Analysis

Within each phase, the multigroup path analysis demonstrated acceptable fit (Phase 0: RMSEA = 0.06, SRMR = 0.05, CFI = 0.91; Phase 1: RMSEA = 0.05, SRMR = 0.05, CFI = 0.95; Phase 2: RMSEA = 0.05, SRMR = 0.05, CFI = 0.95). Furthermore, chi-square difference tests indicated that the multigroup models were a better fit to the data than the full group models (Phase 0: chi-square difference = 80, df = 25, *p* < 0.001; Phase 1: chi-square difference = 171, df = 36, *p* < 0.001; Phase 2: chi-square difference = 127, df = 36, *p* < 0.001).

#### Relationship of Violence Acceptance with Violence Outcomes

Examination of the regression estimates for Phase 0 (Table 3) reveals positive associations with violence outcomes (perpetration and victimization) and both rape myth endorsement and DV acceptance across both groups. Observed regression estimates were higher in the group that did witness parental IPV compared to the group that did not witness parental IPV, as indicated by the CIs for the unstandardized regression betas. This trend was observed across all phases. Examination of the unstandardized regression betas and CIs indicates a pattern for the group that did witness parental IPV, suggesting a strengthening of the relationship between violence acceptance and violence outcomes between phases.

**Table 3:** Path model unstandardized beta estimates (99% CI)

	Phase 0		Phase 1		Phase 2	
Path	Did Not Witness Parental IPV *B* (99% CI *B*)	Did Witness Parental IPV *B* (99% CI *B*)	Did Not Witness Parental IPV *B* (99% CI *B*)	Did Witness Parental IPV *B* (99% CI *B*)	Did Not Witness Parental IPV *B* (99% CI *B*)	Did Witness Parental IPV *B* (99% CI *B*)
Intervention ➔ Training	–	–	1.66** (1.64, 1.69)	1.61** (1.59, 1.64)	1.59** (1.57, 1.62)	1.55** (1.52, 1.58)
Intervention ➔ Psychological DV Perpetration	-.05 (-.13, .02)	-.10 (-.31, .11)	.03 (-.02, .08)	-.03 (-.21, .15)	-.04 (-.09, .01)	-.27* (-.48, -.06)
Intervention ➔ Physical DV Perpetration	-.02 (.00, .05)	.02 (0.06, .09)	.02 (-.01, .04)	.06 (-.02, .14)	-.01 (-.04, .01)	-.10* (-.17, -.02)
Intervention ➔ Psychological DV Victimization	-.07 (-.20, .06)	.02 (-.30, .33)	.06 (-.02, .14)	-.17 (-.43, .09)	-.10* (-.17, -.02)	-.30* (-.58, -.01)
Intervention ➔ Physical DV Victimization	-.01 (-.06, .05)	-.01 (-.11, .09)	.02 (-.01, .05)	-.03 (-.13, .08)	-.02 (-.04, .01)	-.07 (-.17, .03)
Training Received ➔ Rape Myths	.13*^+^ (.01, .27)	.12^+^ (-.14, .38)	-.08** (-.13, -.03)	-.10 (-.23, .03)	-.17** (-.22, -.12)	-.31** (-.47, -.16)
Training Received ➔ DV Acceptance	.10 (-.03, .23)	.15 (-.10, .41)	-.05 (-.10, .01)	-.07 (-.20, .06)	-.15** (-.20, -.10)	-.29** (-.45, -.16)
Rape Myths Psychological DV Perpetration	.03** (.01, .06)	.21** (.15, .26)	.05** (.04, .06)	.28** (.25, .31)	.04** (.03, .05)	.27** (.21, .32)
Rape Myths Physical DV Perpetration	.01** (.00, .01)	.06** (.04, .09)	.01** (.00, .01)	.09** (.06, .11)	.01** (.00, .01)	.10** (.07, .12)
Rape Myths ➔ Psychological DV Victimization	.07** (.04, .09)	.19** (.14, .24)	.06** (.04, .08)	.20** (.15, .25)	.06** (.04, .09)	.26** (.21, .31)
Rape Myths ➔ Physical DV Victimization	.01* (.00, .02)	.06** (.03, .09)	.01** (.01, .02)	.09** (.07, .12)	.01** (.00, .01)	.11** (.09, .14)
DV Acceptance ➔ Psychological DV Perpetration	.07** (.04, .09)	.22** (.17, .30)	.08** (.07, .09)	.25** (.20, .30)	.07** (.06, .08)	.31** (.26, .36)
DV Acceptance ➔ Physical DV Perpetration	.02** (.02, .03)	.09** (.06, .11)	.03** (.03, .04)	.10** (.08, .13)	.02** (.02, .03)	.10** (.07, .13)
DV Acceptance ➔ Psychological DV Victimization	.08** (.05, .10)	.27** (.20, .35)	.11** (.08, .13)	.28** (.23, .34)	.09** (.07, .12)	.31** (.26, .36)
DV Acceptance ➔ Physical DV Victimization	.03** (.02, .04)	.10** (.07, .13)	.03** (.03, .04)	.08** (.06, .11)	.03** (.02, .03)	.09** (.07, .12)

Frequency of violence outcomes were adjusted for demographic controls by including direct paths from sex, sexual orientation, meal status, race, and relationship status to each violence outcome

^*^ = *p* ≤ .01, ** = *p* ≤ .001

+  = reflects the path between intervention and violence acceptance score

– = path not tested

#### Relationships with Intervention in Phases 1 and 2

**Fig. 3:**
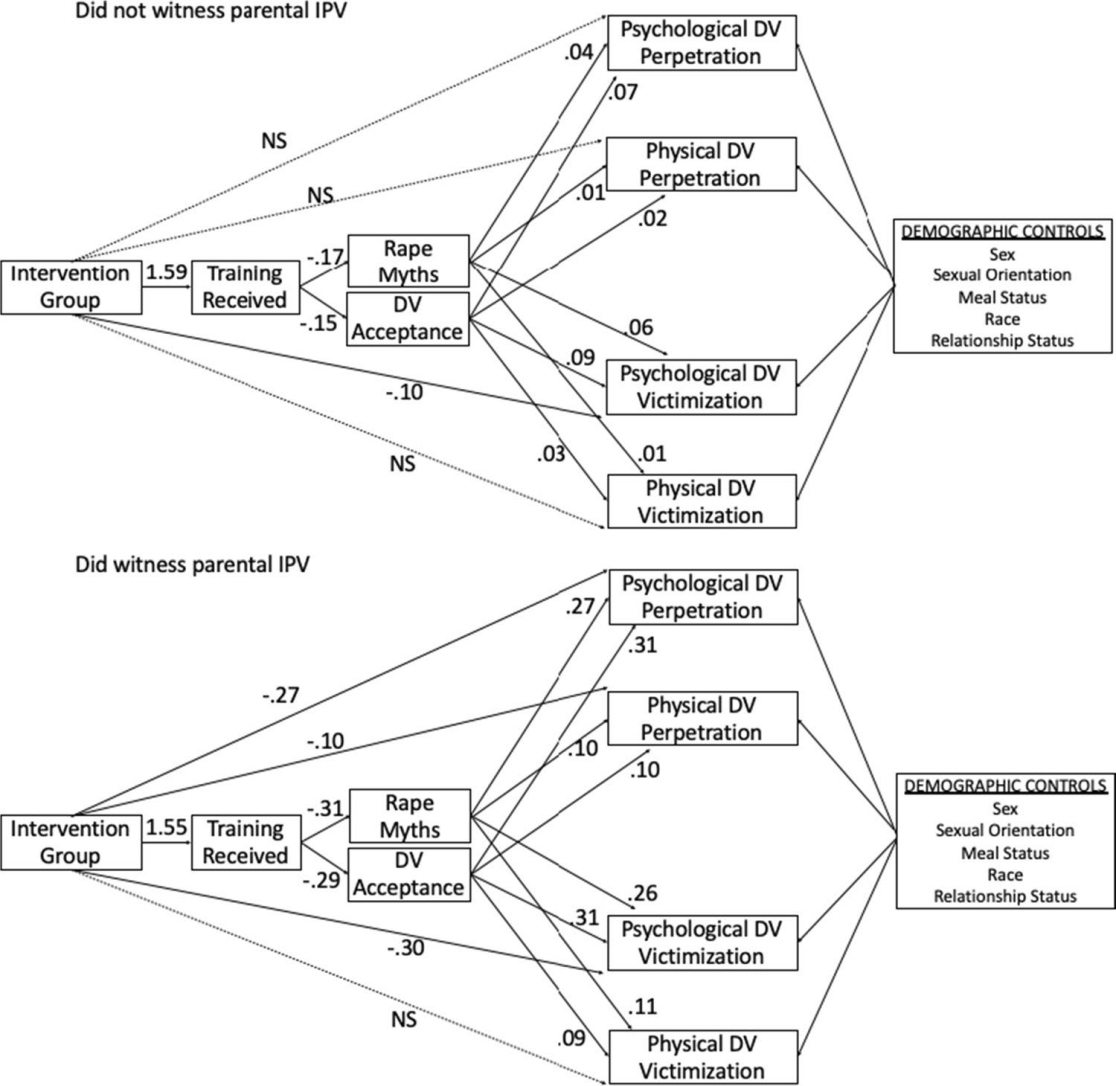
Unstandardized path estimates for model in Phase 2. Note: NS = not significant at *p* < .01

Across Phase 1 and Phase 2 (Table 3 and Fig. 3 for Phase 2), the intervention was positively associated with amount of training received at similar strengths for both exposure groups. Amount of training received was associated with lower endorsement of rape myths for the group who did not witness parental IPV during Phase 1, but this was not observed for the group that did witness parental IPV. Amount of training received was not associated with DV acceptance for either group in Phase 1. In Phase 2, the relationship between rape myths endorsement and DV acceptance was similar across the two groups; more training was associated with lower scores.

The intervention was not associated with violence outcomes during Phase 1 for either exposure group. During Phase 2, the intervention was directly associated with lower reports of psychological DV victimization among those who did not witness parental IPV. In contrast, the intervention was associated with lower reports of psychological DV victimization and perpetration and physical DV perpetration but not physical DV victimization for those who witnessed parental IPV. Examination of the standardized direct, indirect, and total effects of the intervention on violence outcomes (Table 4) indicates that total effects were not significant until Phase 2 of the intervention. During that phase, among those who did not witness parental IPV, significant standardized total effects were observed for the outcomes of physical DV perpetration and psychological DV victimization. During Phase 2 for those who did witness parental IPV, significant total effects were observed for both perpetration outcomes and psychological DV victimization. In general, the magnitudes of these effects were small. The largest effect was observed on psychological DV victimization among those who did not witness parental IPV, followed by perpetration outcomes among those who did witness parental IPV.

**Table 4:** Direct, indirect, and total effects (standardized) of intervention on dependent variables

	Phase 0		Phase 1		Phase 2	
	Did Not Witness Parental IPV	Did Witness Parental IPV	Did Not Witness Parental IPV	Did Witness Parental IPV	Did Not Witness Parental IPV	Did Witness Parental IPV
Outcome	Direct, Indirect, Total Effect	Direct, Indirect, Total Effect	Direct, Indirect, Total Effect	Direct, Indirect, Total Effect	Direct, Indirect, Total Effect	Direct, Indirect, Total Effect
Psychological DV Perpetration	-.019, .004*, -.015	-.018, .010, -.008	.010, -.004, .006	-.004, -.011, -.015	-.014, -.010, -.039	-.039*, -.038*, -.077*
Physical DV Perpetration	-.019, .003*, -.016	.008, .009, .017	.014, -.003, .011	.024, -.010, .014	-.013, -.007*, -.013*	-.036*, -.036*, -.072*
Psychological DV Victimization	-.014, .003*, -.010	.002, .008, .010	.012, -.003, .009	-.019, -.007, -.026	-.020*, -.008*, -.095*	-.033, -.030*, -.063*
Physical DV Victimization	-.004, .002*, -.001	-.003, .008, .005	.012, -.003, .009	-.008, -.008, -.016	-.011, -.010*, -.016	-.039, -.031*, -.054

^*^ = *p* < .01

## Discussion

Answering the first research question, *Green Dot* appears to be more effective at reducing teen DV for high school youth who witnessed parental IPV, as the intervention was associated with a reduction in more forms of teen DV during Phase 2 (including psychological DV victimization and perpetration and physical DV perpetration). Comparatively, the intervention was associated with a reduction in only psychological DV victimization at Phase 2 among youth who did not witness parental IPV. Although we cannot link individuals over time, the analytic approach used herein allowed us to compare paths’ significance, magnitude, and direction between phases among largely the same students to determine when, whether, for whom, and how the intervention works.

The strengths of the unstandardized path coefficients were small, indicating a small, but significant, reduction in the frequency of these forms of DV. During Phase 2 of the intervention, a small percentage of students received a 5-hour skills based training to teach them how to intervene when they saw a concerning situation. It is possible that the direct effect that was observed between intervention and reduction in multiple forms of violence, especially for youth with parental IPV exposure, is a result of this intensive training (supported by the finding that the indirect paths were significant). Trained bystanders likely had the skills to intervene after receiving the training, and the opportunity to intervene more often among their peers who had prior parental IPV exposure. The finding that *Green Dot* was associated with lower levels of teen DV perpetration is an important contribution to the field of violence prevention, as there historically has been an overemphasis on risk reduction techniques that aim to prevent victimization. Additionally, it is important to note that the intervention was not associated with violence reduction until Phase 2, underscoring the need for long-term phased implementation and evaluation of complex interventions.

Prior research has demonstrated that participation in healthy relationship programs can reduce teen DV among individuals who have been exposed to family violence (Reidy et al., 2017; Rizzo et al., 2018; Wolfe, et al., 2003a, b), however this is the first known examination into the efficacy of a universally applied bystander intervention program to reduce teen DV among youth who had previously witnessed parental IPV. While bystander intervention programs, among other primary prevention programs, have been shown to reduce teen DV among high school youth (Coker et al., 2017; Edwards et al., 2019; Foshee et al., 2005; Munoz-Frenandez et al., 2019; Wolfe et al., 2009), this research indicates that a key component of that change is through changing rates of violence perpetration among the high-risk group of youth who have been exposed to family violence.

Answering the second research question, results indicate that *Green Dot* works to reduce teen DV through reducing endorsement of rape myths and acceptance of DV during Phase 2. For both exposure groups at Phase 2, amount of training received was associated with lower violence acceptance scores. Because Phase 2 consists of the more intensive training sessions, it is possible that this training setting provided a more focused attention on busting myths and challenging attitudes and stereotypes related to violence acceptance, partially explaining why the direct path between amount of training received and violence acceptance was by and large not observed until Phase 2. In turn, across phases for both exposure groups, violence acceptance scores were associated with DV outcomes. While significant for both groups, the strength of these associations was higher for the students who did witness parental IPV. Exposure to family violence is a robust predictor of future IPV victimization (Park & Kim, 2018), thus it is important to understand the mechanism through which this relationship exists. In alignment with past research (Lichter & McCloskey, 2004), violence acceptance was found to be strongly associated with DV victimization/perpetration among those who have been exposed to family violence. Acceptance of violence has already been shown to mediate the effectiveness of *Green Dot* to reduce sexual violence perpetration at the school-level (Bush et al., 2019), and this research further extends these findings by demonstrating this path also operates at the individual-level, is significant for teen DV, and is moderated by prior exposure to family violence. Additionally, reduction in violence acceptance was not observed until Phase 2 of the intervention, again underscoring the need for long-term intervention implementation and evaluation.

Overall, this research suggests that being assigned to an intervention school does reduce DV victimization and perpetration, especially among youth who were exposed to parental IPV. However, the findings also suggest that a significant explanation for this result is because of indirect paths (such as between training received and violence acceptance). Attending a school where *Green Dot* is being implemented may slightly reduce victimization and perpetration of teen DV among youth who were exposed to parental IPV (possibly because peers are using bystander behaviors to prevent violence acts from occurring). But exposing these high-risk youth directly to training (the more the better) can challenge their own levels of violence acceptance, which in turn can reduce their own DV victimization and perpetration. While the goal of *Green Dot* is to train bystander to prevent perpetration from occurring, other innovators of bystander intervention aimed to approach men as potential bystanders because of the belief that it would be less threatening than approaching men as potential perpetrators (Katz, 2018). One explanation for the current findings is that participation in bystander training programs does indeed directly reduce perpetration of the participants, beyond the effect that being in an intervention school and being the recipient of bystander actions from others might have alone. Of course, it is also possible and likely that other factors mediate the relationship between intervention and violence outcomes, which were not assessed for in the current investigation.

It is clear from this sample that a major driver of DV within high schools is from students who witnessed parental IPV. It is encouraging that *Green Dot*, a program which was designed to be a universal prevention program of sexual violence, works to prevent multiple forms of teen DV, including DV perpetration, for this high-risk group. This research identified that acceptance of violence is highly influential to understanding teen DV victimization and perpetration among youth who previously witnessed parental IPV, and also indicates that receiving training is an effective way to reduce the amount of violence acceptance. To further enhance the effectiveness of bystander programs, interventions should continue to aim to reduce violence acceptance. Doing so can lead to further reductions in teen DV for all youth, but especially among the high-risk group of youth who had previously witnessed parental IPV.

### Limitations

A limitation of this investigation is the measurement of the primary exposure variable – witnessing parental IPV. This construct was captured using a single-item indicator, which did not allow for a more thorough investigation into childhood exposure to IPV, including being the target of violence by parental figures and other adverse childhood experiences. The reliability and validity of a single-item indicator is unable to be ascertained, and using frequency count rating scales to assess long periods of recall is often inaccurate. As such, for this investigation, to create the exposure groups, we dichotomized the witnessing parental IPV variable and did not rely on the frequency counts. The use of self-report data threatens the internal validity of study findings, especially in intervention schools. Individuals from the intervention schools may have been motivated to minimize their report of exposure to teen DV. In addition, compensatory rivalry or social desirability bias also threaten the validity of the findings, as individuals from the intervention schools may have downplayed their exposure to teen DV because they knew they were part of the intervention and wanted to make their school look favorable. Relatedly, there may be bias in our model due to our decision to include training received as a mediator rather than a moderator. There is likely bias in the findings as a result of the decision to use case-wise deletion instead of multiple imputation. However, unknowable bias is introduced when imputing the values of victimization/perpetration experiences. Therefore we decided to interpret the results in light of the known biases caused by these missing data over the unknown biases caused by imputing. Based on the findings that a greater proportion of survey non-completers were in higher grades, received a free or reduced-priced meal, experienced family abuse, and were male, non-White, and not exclusively heterosexual, it is likely that these results do not generalize to the most marginalized high school youth. Additionally, study results are likely not generalizable to all high school students in the United States, as the study sample consisted only of students in Kentucky, which has unique culture, values, and politics that are not representative of all parts of the United States; however, results may be generalizable to other regions in the South. Finally, the data are relatively dated, with data collection beginning in 2010. Thus, the results may not be fully applicable to high school youth today.

## Conclusions

*Green Dot* is considered to be a universal primary prevention bystander intervention program to reduce interpersonal violence. This investigation revealed that the *Green Dot* intervention largely works by changing amount of violence acceptance at Phase 2, which is strongly associated with teen DV among youth who had previously witnessed parental IPV. Furthermore, the intervention was associated with a reduction in more forms of violence for this high-risk group during Phase 2, including psychological DV victimization and perpetration and physical DV perpetration, compared to the student who did not witness parental IPV who only saw reductions in psychological DV victimization. As bystander intervention programs continue to refine their techniques and hone in on mechanisms for change, it is imperative that they consider the unique needs of high-risk individuals who previously have been exposed to violence. Doing so can enhance the effectiveness of these programs, thereby reducing the rates of teen DV.
